# Hippocampal-Prefrontal Interactions in Cognition, Behavior and Psychiatric Disease

**DOI:** 10.3389/fnsys.2015.00190

**Published:** 2016-01-26

**Authors:** Torfi Sigurdsson, Sevil Duvarci

**Affiliations:** Institute of Neurophysiology, Neuroscience Center, Goethe University FrankfurtFrankfurt, Germany

**Keywords:** hippocampus, prefrontal cortex, synchrony, functional connectivity, schizophrenia, animal models of mental disorders

## Abstract

The hippocampus and prefrontal cortex (PFC) have long been known to play a central role in various behavioral and cognitive functions. More recently, electrophysiological and functional imaging studies have begun to examine how interactions between the two structures contribute to behavior during various tasks. At the same time, it has become clear that hippocampal-prefrontal interactions are disrupted in psychiatric disease and may contribute to their pathophysiology. These impairments have most frequently been observed in schizophrenia, a disease that has long been associated with hippocampal and prefrontal dysfunction. Studies in animal models of the illness have also begun to relate disruptions in hippocampal-prefrontal interactions to the various risk factors and pathophysiological mechanisms of the illness. The goal of this review is to summarize what is known about the role of hippocampal-prefrontal interactions in normal brain function and compare how these interactions are disrupted in schizophrenia patients and animal models of the disease. Outstanding questions for future research on the role of hippocampal-prefrontal interactions in both healthy brain function and disease states are also discussed.

## Introduction

The brain is organized into multiple areas with relatively specialized functions yet in order to generate adaptive behavior, neural activity must be coordinated and integrated across distributed brain regions (Varela et al., 2001; Bressler and Menon, 2010). The last decade has witnessed important advances in our understanding of how such inter-areal interactions underlie various behaviors and cognitive functions. Studies in both animals and human subjects have revealed correlations in neural activity between brain regions that often change dynamically with various task demands (Gregoriou et al., 2009; Harris and Gordon, 2015). Complementing these neural activity-based measures are anatomical methods that allow the long-range projections that might support such inter-areal interactions to be examined in the intact brain (Assaf and Pasternak, 2008; Chung and Deisseroth, 2013). Optogenetic tools have also been used to manipulate long-range projections and test their contributions to inter-areal interactions and behavior (Tye and Deisseroth, 2012). Studies using the above methods have greatly advanced our understanding of how the brain operates as a large-scale network to support diverse sensory, cognitive and behavioral functions. At the same time, it has also become clear that a breakdown in inter-regional coordination is likely an important pathophysiological mechanism in various psychiatric diseases such as schizophrenia and autism, as well as in neurological disorders such as Parkinson’s and Alzheimer’s disease (Uhlhaas and Singer, 2006, 2012; Pettersson-Yeo et al., 2011). The detailed neural circuit disruptions underlying these interaction abnormalities and how they relate to the various risk factors for psychiatric disease have also begun to be investigated in animal models of psychiatric illness (Sigurdsson, 2015; Spellman and Gordon, 2015).

In recent years, interactions between the hippocampus and prefrontal cortex (PFC) have emerged from animal studies as playing a key role in various cognitive and behavioral functions (Benchenane et al., 2011; Colgin, 2011; Harris and Gordon, 2015). Disruptions in hippocampal-prefrontal interactions have also been observed in psychiatric disease, most notably schizophrenia (Tost et al., 2012; Godsil et al., 2013) and have also been reported in animal models of the illness (Sigurdsson, 2015). The goal of this review is to summarize and discuss these findings in order to provide an overview of the role of hippocampal-prefrontal interactions in health and disease. We begin by discussing the anatomical and functional organization of the hippocampus and PFC and their anatomical interconnections. Because the studies discussed in this review encompass rodents and primates, the comparative aspects of the hippocampal-prefrontal circuit are also discussed. The methods used to measure interactions between the two structures are also briefly reviewed. We then review findings from studies that have related hippocampal-prefrontal interactions to specific cognitive functions in rodents, primates and humans. The results of studies examining hippocampal-prefrontal interactions in schizophrenia patients and animal models of the illness then follows. Finally, we conclude by discussing emerging themes in studies of hippocampal-prefrontal interactions and outline outstanding questions for future research.

## Anatomical Organization of the Hippocampus, Prefrontal Cortex and Their Interconnections

The hippocampus is a medial temporal lobe structure found in all mammalian species that plays a key role in spatial navigation as well as several forms of learning and memory (Buzsáki and Moser, 2013; Eichenbaum and Cohen, 2014; Gruart et al., 2015). A major source of its inputs comes from the entorhinal cortex, which links the hippocampus with the rest of the neocortex (Moser et al., 2010). The hippocampus is organized into several subfields, including the dentate gyrus (DG) and cornum ammonis areas 1 (CA1) and three (CA3). It is also organized along a longitudinal axis which in primates, including humans, extends from an anterior to a posterior pole. In rodents, the longitudinal axis extends between the dorsal and ventral poles of the hippocampus which are homologous with the posterior and anterior poles, respectively, in the primate (Fanselow and Dong, 2010; Strange et al., 2014). There is compelling evidence that a gradient exists along the longitudinal axis with respect to gene expression and anatomical connectivity, as well as physiological and behavioral functions (Fanselow and Dong, 2010; Strange et al., 2014). For example, the activity of many hippocampal neurons in rodents (so-called “place cells”) represent the animal’s current location in its environment and the precision of their spatial representation increases as one moves from the ventral to the dorsal pole (Kjelstrup et al., 2008). Lesion studies also suggest that the functions classically associated with the hippocampus—the processing of spatial information and memory—are largely subserved by its dorsal subregion whereas the ventral hippocampus (vHPC) is more involved in emotional and motivational behaviors such as anxiety (Bannerman et al., 2004). This functional dissociation is also reflected in the unique anatomical connections of the dorsal and ventral poles to both afferent and efferent structures (Fanselow and Dong, 2010; Strange et al., 2014).

In contrast to the hippocampus, the PFC is a more phylogenetically divergent structure. It is most prominent in primates, especially humans, and is critical for higher-order cognitive processes and emotional regulation. The primate PFC is organized into several subregions but can be broadly separated into a dorsolateral division that is involved in cognitive functions such as executive control, attention and working memory, and a ventromedial (or orbitomedial) division more involved in emotional and motivational regulation (Koenigs and Grafman, 2009; Fuster, 2015). In rodents the PFC can be defined, like in primates, as the cortical region receiving its main thalamic input from the mediodorsal thalamus (Uylings and van Eden, 1990). The rodent PFC is typically divided into medial, lateral and ventral subdivisions, each of which in turn consist of several subregions (Uylings and van Eden, 1990; Heidbreder and Groenewegen, 2003; Hoover and Vertes, 2007). Lesion studies have shown the rodent medial PFC (mPFC) to be involved in some of the cognitive functions attributed to the primate PFC including working memory, attentional set-shifting and the regulation of emotional responses (Vertes, 2006; Kesner and Churchwell, 2011; Euston et al., 2012). There is also evidence supporting a more general distinction between the dorsal mPFC, which is involved in cognitive functions, and the ventral mPFC which is more involved in emotional behaviors (Heidbreder and Groenewegen, 2003). The majority of rodent studies that will be discussed below have focused on the dorsal mPFC. Based on the functional roles of different mPFC subdivisions (as well as their anatomical connections) it has been suggested that the rodent dorsal mPFC has features of dorsolateral primate PFC whereas the ventral mPFC is more similar to the primate orbitomedial PFC (Uylings and van Eden, 1990; Condé et al., 1995; Hoover and Vertes, 2007; Churchwell et al., 2010). Nevertheless, because of the unique phylogenetic development of the PFC, it is difficult to draw exact homologies between its subregions in rodents and primates (Preuss, 1995).

**Figure 1:**
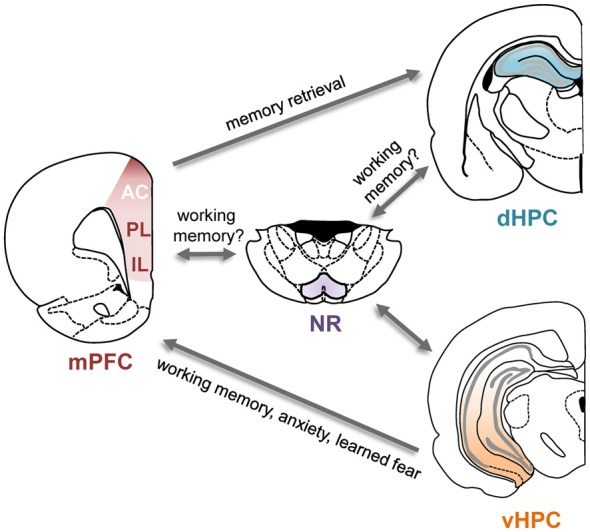
**Direct and indirect connections between the prefrontal cortex (PFC) and hippocampus and their functional roles.** Schematic of the direct and some of the indirect connections between the hippocampus and PFC. Arrows indicate direction of the projections. Functional roles of these projections are indicated in the text next to the arrows; for details and supporting references, see main text. AC, anterior cingulate cortex; dHPC, dorsal hippocampus; IL, infralimbic cortex; mPFC, medial prefrontal cortex; NR, nucleus reuniens; PL, prelimbic cortex; vHPC, ventral hippocampus.

Several direct and indirect anatomical pathways link the hippocampus and the PFC (Figure 1). In both rodents and primates, the PFC receives monosynaptic projections from the hippocampus (Barbas and Blatt, 1995; Condé et al., 1995; Hoover and Vertes, 2007). These projections originate almost exclusively in the vHPC and primarily target the mPFC, with some evidence suggesting stronger projections to ventral subregions (Rosene and Van Hoesen, 1977; Barbas and Blatt, 1995; Condé et al., 1995; Hoover and Vertes, 2007). Electrically stimulating the vHPC elicits short-latency excitatory synaptic responses in the mPFC, indicative of a glutamatergic projection (Thierry et al., 2000). A monosynaptic projection from the PFC to the dorsal hippocampus (dHPC) has also been identified recently in the mouse (Rajasethupathy et al., 2015). This projection originates in the anterior cingulate (AC) subdivision of the mPFC and terminates in the CA1 and CA3 subfields of the dHPC. In addition to these monosynaptic connections, bidirectional interactions between the two structures could also be achieved via several indirect routes. One possible relay is the nucleus reuniens (NR) of the thalamus which is reciprocally connected to both the dorsal and vHPC as well as the mPFC (Vertes, 2006; Cassel et al., 2013). The lateral EC is also reciprocally connected with the PFC as well as the hippocampus (Moser et al., 2010).

## Measuring Hippocampal-Prefrontal Interactions

The anatomical connections between the hippocampus and the PFC, reviewed in the previous section, enable the two structures to interact and influence each other. Various approaches have been used to examine hippocampal-prefrontal interactions in the studies that will be discussed below and are worth reviewing briefly before proceeding (also see Table 1). Generally speaking, interactions between brain regions are measured by asking whether fluctuations in their neuronal activity are correlated in time. In animal studies, such correlations are typically derived from electrophysiological recordings and are referred to as measures of “neural synchrony”. The most direct method for measuring inter-areal synchrony is to compute the cross-correlation of neuronal spike trains in two regions. This approach has revealed that hippocampal and prefrontal neurons often spike within a short time (~100 ms) of each other (Jones and Wilson, 2005a; Siapas et al., 2005). Furthermore, spikes in prefrontal neurons can lead or lag behind spikes in the hippocampus, revealing the directionality of influence between the two regions (Siapas et al., 2005; Wierzynski et al., 2009). Many prefrontal neurons are also modulated by the phase of hippocampal theta oscillations, which are rhythmic 4–12 Hz fluctuations in the local field potential (LFP) that are reliably observed in the hippocampus of behaving rodents (Buzsáki, 2002). Prefrontal neurons tend to fire more at certain phases of the theta oscillation, a phenomenon known as “phase locking” (Hyman et al., 2005; Siapas et al., 2005; Sigurdsson et al., 2010). Prefrontal neurons that are phase-locked to hippocampal theta oscillations also tend to show cross-correlations with hippocampal neurons, suggesting that the two measures reflect the same underlying phenomenon (Siapas et al., 2005). Interestingly, prefrontal neurons can be phase-locked more strongly to either past or future phases of hippocampal theta oscillations although on average phase-locking to the past is stronger (Jones and Wilson, 2005a; Siapas et al., 2005; Sigurdsson et al., 2010), perhaps reflecting the direct monosynaptic influence of the hippocampus on the PFC. Finally, hippocampal-prefrontal synchrony can be observed in the “coherence” of LFPs recorded in the two structures, which measures the consistency of their phase differences over time (Jones and Wilson, 2005a; Sigurdsson et al., 2010), as well as in correlated fluctuations in their amplitude, or “power correlations” (Adhikari et al., 2010). LFPs reflect, in large part, the synaptic currents (driven by both local and long-range inputs) from neurons in the vicinity of the recording electrode (Buzsáki et al., 2012). However, LFPs can also be volume conducted from regions that are distant to the electrode. This is an important caveat of measures of synchrony based solely on LFPs, which should ideally be complemented by measures based on neuronal spiking (Buzsáki et al., 2012). Measures of phase-locking, coherence and power correlations can be obtained for different frequency bands. Because of the prominence of theta (4–12 Hz) oscillations in the hippocampus, hippocampal-prefrontal synchrony is frequently reported in this range, but can also be seen at other frequencies, such as beta (12–30 Hz) and gamma (30–80 Hz). It is worth noting that although most studies have examined synchrony between the PFC and the dHPC, synchrony with the vHPC is stronger (Adhikari et al., 2010; O’Neill et al., 2013), likely reflecting the monosynaptic prefrontal projections from this hippocampal subregion.

**Table 1:** **Overview of methods used to measure interactions between brain regions**.

Method	Definition
Cell cross-correlations	Measures the degree to which neurons in two structures fire spikes at the same time
Phase locking	Measures how the firing of neurons in one region is modulated by the phase of LFP oscillations in another region
Coherence	Measures whether the phase relationship of LFP oscillations in two structures is consistent over time
Power correlations	Measures whether fluctuations in the power of LFP oscillations in two structures are correlated over time
Functional connectivity	Measures whether fluctuations in the fMRI BOLD signal in two brain regions are correlated over time

Although hippocampal-prefrontal synchrony has mostly been examined in rodents, it can also be observed in non-human primates (Brincat and Miller, 2015) and humans (Axmacher et al., 2008). However, for methodological reasons a more common approach in human studies is to measure hippocampal-prefrontal interactions using the blood oxygen-level-dependent (BOLD) signal derived from functional magnetic resonance imaging (fMRI). The most straightforward approach is to correlate fluctuations in the BOLD signal across brain regions, a measure commonly known as “functional connectivity”. More advanced analytical methods can also measure “effective connectivity”, which quantifies the causal influence of one area over another, as well as how connectivity varies with task variables (Fornito and Bullmore, 2012; Buckner et al., 2013). Connectivity measures can take on both positive values (i.e., reflecting positive correlations in the BOLD signal) as well as negative values (reflecting anti-correlations). In subjects at rest, positive connectivity is frequently observed among anatomically connected regions and is thought to reflect the “intrinsic” connectivity of the brain (Buckner et al., 2013). For example, the hippocampus shows positive functional connectivity with the mPFC in humans (Vincent et al., 2006), consistent with the anatomy. In contrast, negative connectivity is thought to occur between functionally antagonistic brain regions, although it may also reflect the particular analysis methods used (Buckner et al., 2013). It is worth emphasizing that functional connectivity measures neuronal correlations at a vastly different timescale than electrophysiological measures of neural synchrony. Because of the slow time course of the BOLD signal, functional connectivity can only quantify correlations occurring at frequencies below 0.1 Hz (Fox and Raichle, 2007) whereas electrophysiological recordings typically measure synchrony at frequencies between 1 and 100 Hz. This point is important to keep in mind when comparing human and animal studies and one which we will return to later.

## The Role of Hippocampal-Prefrontal Interactions in Cognition and Behavior

As reviewed in the previous section, electrophysiological studies have revealed that neural activity in the hippocampus and PFC is often correlated or synchronized in time, indicative of interactions between the two structures. What is the functional role of these interactions? The answer to this question in part lies in considering the individual functions of the hippocampus and PFC and these are worth reviewing briefly, although a comprehensive survey is beyond the scope of this review. As mentioned previously, the hippocampus has primarily been associated with spatial navigation as well as the formation and storage of long-term memories (reviewed in Buzsáki and Moser, 2013; Eichenbaum and Cohen, 2014; Gruart et al., 2015). It has also been suggested that the spatial and mnemonic functions are manifestations of a more general role of the hippocampus in representing the relationship between objects and events in both space and time (Eichenbaum and Cohen, 2014). In addition to these cognitive functions of the hippocampus, its ventral pole plays a role in emotional behaviors such as fear and anxiety (reviewed in Bannerman et al., 2004). The PFC has classically been viewed as being involved in “executive” functions such as decision making, working memory and attentional set shifting (Miller and Cohen, 2001; Kesner and Churchwell, 2011; Fuster, 2015). It is also important for long-term memories (Euston et al., 2012) as well as the regulation of emotional responses (Öngür and Price, 2000; Heidbreder and Groenewegen, 2003; Koenigs and Grafman, 2009), in particular fear and anxiety (Tovote et al., 2015).

**Figure 2:**
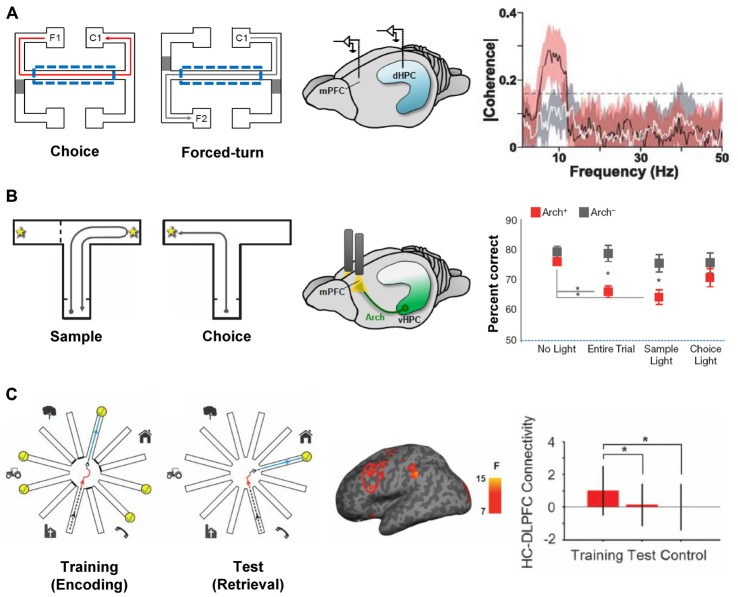
**Hippocampal-prefrontal interactions during spatial working memory (SWM). (A)** Hippocampal-prefrontal synchrony during a SWM task. Left: during “choice” trials, rats had to choose the arm on the same side as the one they left from (i.e., C1 when leaving from F1) whereas during “forced-turn” trials animals were directed to enter one arm by closing off the other with a barrier. Working memory is therefore required in the center arm (dashed line) during choice trials but not forced-turn trials. Simultaneous recordings from the dHPC and mPFC (middle) revealed stronger theta coherence between the two structures during center arm runs in choice compared to forced-run trials (right). Adapted from Jones and Wilson (2005a). **(B)** Silencing ventral hippocampal inputs to the PFC impairs SWM performance. Left: mice performed a SWM task where, in the “choice” phase, they had to choose the arm opposite to the one visited in the “sample” phase. During task performance, ventral hippocampal inputs to the PFC were optogenetically silenced with light (middle) during different task phases. Performance in the choice phase was only impaired when silencing was performed during the entire trial or in the sample phase (right). Adapted from Spellman et al. (2015). **(C)** Hippocampal-prefrontal functional connectivity in human subjects during a virtual reality SWM task. Left: during the “training” phase subjects navigated a virtual reality version of the radial arm maze and had to collect reward from six arms (yellow) while the other six were closed. During the “test” phase subjects had to enter the previously closed arms to collect reward. Functional connectivity measured between the dorsolateral PFC and hippocampus (middle) was strongest during the training phase (right). Adapted from Bähner et al. (2015).

Hippocampal-prefrontal interactions are likely to be important during behaviors in which both structures are involved and when their different functions need to be coordinated. One example of such a behavior is spatial working memory (SWM), which measures animals’ ability to remember spatial locations in the short-term. In a typical SWM task, animals must choose which arm of a maze to enter during a “choice phase” based on their memory of which arm they visited a short while ago during the “sample phase” (Dudchenko, 2004). Lesion studies have shown that SWM tasks require the integrity of both the hippocampus and the PFC in rodents (Yoon et al., 2008; Churchwell and Kesner, 2011; Kesner and Churchwell, 2011) and a number of electrophysiological studies have examined hippocampal-prefrontal interactions during performance of such tasks. In a seminal study, Jones and Wilson (2005a) recorded simultaneously from the two structures in rats and observed increased hippocampal-prefrontal synchrony in the choice phase (from phase-locking, LFP coherence and cell pair cross-correlations) compared to a task phase in which the overt behavior was the same but no working memory was required (Figure 2A). The increase in synchrony was restricted to the theta frequency range and was absent when animals made a wrong choice, further supporting its role in behavioral performance. These findings were subsequently replicated and extended in several studies (Hyman et al., 2010; Sigurdsson et al., 2010; O’Neill et al., 2013; Spellman et al., 2015). Sigurdsson et al. (2010) observed that hippocampal-prefrontal theta synchrony gradually increased in mice during the learning of a SWM task in parallel with improved behavioral performance, suggesting that plasticity in this circuit might underlie task acquisition. The increase in hippocampal-prefrontal synchrony during the choice phase of a SWM task was also observed for both the dorsal and ventral poles of the hippocampus (O’Neill et al., 2013). In contrast to the increase in hippocampal-prefrontal theta synchrony during the choice phase, Spellman et al. (2015) observed increased gamma synchrony during the sample phase, when animals presumably need to encode to-be-remembered information. The increase in synchrony was predictive of behavioral performance, suggesting that hippocampal-prefrontal interactions in different frequency bands might differentially support the encoding and retrieval or maintenance of information in SWM tasks.

Hippocampal-prefrontal functional connectivity has also been examined during working memory in human subjects in several studies, all of which have focused on connectivity with the dorsolateral PFC. In one notable study subjects navigated a virtual reality version of the radial arm maze, a SWM task that is commonly used in rodents (Bähner et al., 2015; Figure 2C). The authors found that hippocampal-prefrontal functional connectivity was strongest during the sample phase of the task and that the strength of connectivity correlated with SWM (but not visual working memory) performance. The results of Bähner et al. (2015) therefore suggest that the hippocampal-prefrontal circuit is recruited during SWM in both rodents and humans. Hippocampal-prefrontal interactions have also been observed during working memory for other stimulus domains. One noteworthy study recorded neural activity directly from the hippocampus and PFC in a patient undergoing surgery for epilepsy. The authors found that during a facial working memory task, hippocampal-prefrontal synchrony became stronger as the working memory load (the number of to-be-remembered items) increased (Axmacher et al., 2008). Other studies, using fMRI, have also observed increased functional connectivity with increasing working memory load, using either a working memory task for faces (Rissman et al., 2008) or letters (Finn et al., 2010). However, decreases in hippocampal-prefrontal functional connectivity have also been reported with increasing working memory load (Meyer-Lindenberg et al., 2005; Axmacher et al., 2008).

Whereas the studies discussed above provide correlative evidence for the role of hippocampal-prefrontal interactions in working memory they do not address their causal contribution to behavioral performance. Early attempts to address this question in rodents used so-called “disconnection lesions” in which the hippocampus is lesioned (or inactivated) in one hemisphere and the PFC in the opposite hemisphere. Because the connections between the two structures are ipsilateral, such a lesion prevents communication between the remaining hippocampus and PFC. Compared with lesions in which both structures are lesioned on the same side (which allows the two structures to communicate and typically does not have a behavioral effect), disconnection lesions cause impairments in SWM performance (Floresco et al., 1997; Wang and Cai, 2006; Churchwell et al., 2010), consistent with a role for hippocampal-prefrontal interactions in these tasks. These findings have recently been extended and refined using optogenetic methods. Spellman et al. (2015) expressed the neuronal silencer Archaerhodopsin (Arch) in ventral hippocampal neurons and used optical stimulation to silence their synaptic terminals in the mPFC of mice performing a SWM task. This approach made it possible to silence hippocampal afferents with high temporal precision, allowing the contribution of hippocampal-prefrontal interactions to the different phases of the SWM task to be dissected. Interestingly, silencing during the sample phase, but not the choice phase, impaired working memory performance (Figure 2B). Silencing of hippocampal-prefrontal inputs also impaired gamma synchrony during the sample phase, as well as the ability of prefrontal neurons to encode spatial locations during this phase (Spellman et al., 2015). In contrast, hippocampal-prefrontal theta synchrony, which was previously shown to be enhanced during the “choice” phase of SWM tasks (Jones and Wilson, 2005a; Sigurdsson et al., 2010) was not affected by this manipulation.

It is important to note that hippocampal-prefrontal interactions are also observed in spatial tasks that do not explicitly require working memory. In the study of Benchenane et al. (2010), rats had to switch between different rules in order to obtain reward in a Y-maze. Hippocampal-prefrontal synchrony was greatest at the choice point in the maze, in particular following the acquisition of a new rule, and was accompanied by the emergence of cell assemblies among PFC neurons. Similarly, in rats performing a series of sequential decisions in a “wagon-wheel” maze, coherence between hippocampus and PFC (AC subdivision) in the low theta range (5–7 Hz) was highest at choice points, which was also the time when hippocampal and PFC neurons carried most information about animals’ trajectories (Remondes and Wilson, 2013). During a SWM task, hippocampal-prefrontal synchrony was also highest as animals approached the decision point (Jones and Wilson, 2005b). These results suggest that hippocampal-prefrontal interactions may play a more general role in decision-making based on spatial information (Yu and Frank, 2015).

Hippocampal-prefrontal interactions also likely play an important role in long-term memory. A recent study in monkeys performing a declarative memory task (paired-associate learning) revealed hippocampal-prefrontal synchrony that was specific to trial outcome: whereas theta synchrony was seen following errors, synchrony in the alpha/beta range (9–16 Hz) was observed following correct trials (Brincat and Miller, 2015). Hippocampal-prefrontal interactions during sleep have also been hypothesized to support the consolidation of long-term memories and their transfer to the neocortex. Consistent with this view, hippocampal-prefrontal synchrony is observed in sleeping rats, with hippocampal activity leading activity in the PFC (Wierzynski et al., 2009). Patterns of neural activity in the PFC that occur during waking experience are also “replayed” during subsequent sleep at the same time as so-called “sharp-wave ripple” events in the hippocampal LFP (Peyrache et al., 2009). Studies in both humans and non-human primates furthermore suggest that the PFC mediates the strategic retrieval of memories based on behavioral context, by exerting top-down control over the hippocampus and associated medial temporal lobe structures (Simons and Spiers, 2003; Preston and Eichenbaum, 2013). Further supporting this view is the recent identification of a direct projection from PFC to the hippocampus which optogenetic manipulations have shown are both necessary and sufficient for retrieval of a spatial memory, as measured using contextual fear responses (Rajasethupathy et al., 2015).

In addition to the cognitive functions reviewed above, hippocampal-prefrontal interactions are also involved in motivational and emotional behaviors. Theta synchrony between the PFC and the vHPC, but not the dHPC, increases in anxiogenic environments such as the elevated plus maze (Adhikari et al., 2010), consistent with the selective role of the vHPC in emotional behavior (Bannerman et al., 2004). Interestingly, ventral hippocampal-prefrontal synchrony is strongest during periods of safety (i.e., in the closed arms of the elevated plus maze), suggesting that it might play a role in the inhibition of exploratory behavior. However, another study found that silencing the vHPC increased the firing of prefrontal pyramidal neurons (by decreasing firing of interneurons) as well as the expression of conditioned fear following extinction (Sotres-Bayon et al., 2012), suggesting that the ventral hippocampal inputs to the PFC inhibit fear responding. Furthermore, long-term synaptic plasticity at ventral hippocampal inputs to the PFC is involved in the extinction of fear responses (Hugues et al., 2006; Peters et al., 2010). Finally, it is worth emphasizing that both the hippocampus and PFC depend on their interactions with other structures, notably the amygdala, to adaptively control fear responses (Maren et al., 2013; Tovote et al., 2015). During sleep following fear conditioning, synchrony between the amygdala and both the hippocampus and PFC correlates with the consolidation of fear memories (Popa et al., 2010). Synchrony between the PFC and the amygdala is also associated with the successful inhibition of fear, suggesting top-down control over fear responses (Lesting et al., 2013; Likhtik et al., 2014).

## Hippocampal-Prefrontal Interactions in Schizophrenia

The studies reviewed in the previous section demonstrate that the hippocampal-prefrontal circuit plays an important role in various cognitive and emotional functions. There is also considerable evidence that this circuit is disrupted in psychiatric illness (Godsil et al., 2013). Particular attention has been paid to hippocampal-prefrontal interactions in schizophrenia, which we will focus on in this review. Schizophrenia is a devastating psychiatric illnesses that affects roughly one percent of the population and typically emerges between 18 and 25 years of age. The disease manifests itself in numerous symptoms that are classified as either “positive” (hallucinations and delusions, disorganized speech and behavior), or “negative” (flattened affect, social withdrawal, avolition and anhedonia). In addition to positive and negative symptoms, patients display cognitive deficits (including impaired attention, working memory, executive function and verbal memory), which are increasingly seen as critical for daily life functioning (Mesholam-Gately et al., 2009). Although some of the symptoms (particularly the positive ones) can be controlled with drug treatment there exists no cure for schizophrenia and as a result the disease places an enormous burden on patients, their families and society as a whole. A detailed understanding of the causes of the illness is therefore critical for the development of more effective treatment strategies.

To date, a number of brain abnormalities have been identified in schizophrenia patients, ranging from alterations in the structure of neurons to abnormalities in neurotransmitter signaling to disruptions in large-scale brain networks (Meyer-Lindenberg, 2010; Volk and Lewis, 2010; Uhlhaas and Singer, 2012). The hippocampus is among one of the brain regions whose dysfunction has consistently been implicated in the pathophysiology of the disease. The volume of the hippocampus, in particular its anterior pole, is decreased in patients and it is also more metabolically active (Heckers and Konradi, 2010; Small et al., 2011). A recent study in individuals at risk for developing schizophrenia found that the hypermetabolism precedes the reduction in hippocampal volume and also predicts transition to the illness (Schobel et al., 2013). Research in animal models suggests that hyperactivity in the hippocampus could be responsible for the increased dopamine release observed in patients, which in turn is thought to cause the positive symptoms of the disease (Lodge and Grace, 2009; Winton-Brown et al., 2014; Modinos et al., 2015). fMRI studies have also revealed abnormal activation of the hippocampus during memory tasks (Heckers and Konradi, 2010).

The PFC, in particular its dorsolateral subregion, underlies many of the cognitive functions that are disrupted in schizophrenia and has therefore been a major focus of research into the pathophysiology of the disease (Minzenberg et al., 2009; Barch and Ceaser, 2012). Postmortem studies have revealed alterations in the structure and connectivity of both excitatory and inhibitory neurons, including parvalbumin-expressing (PV) interneurons, within the PFC of patients (Volk and Lewis, 2010). Abnormalities in PV interneurons have received a great deal of attention because of their role in the generation of gamma oscillations (reviewed in Buzsáki and Wang, 2012), which are disrupted in schizophrenia patients and are believed to contribute to their cognitive impairments (Uhlhaas and Singer, 2012). fMRI studies have also revealed reduced activation of the dorsolateral PFC, among other brain regions, during tasks requiring cognitive functions such as working memory and executive control (Glahn et al., 2005; Minzenberg et al., 2009; Barch and Ceaser, 2012). There is also increasing evidence that functional connectivity between the dorsolateral PFC and other brain regions, including the hippocampus, is altered (Liang et al., 2006; Pettersson-Yeo et al., 2011).

**Figure 3:**
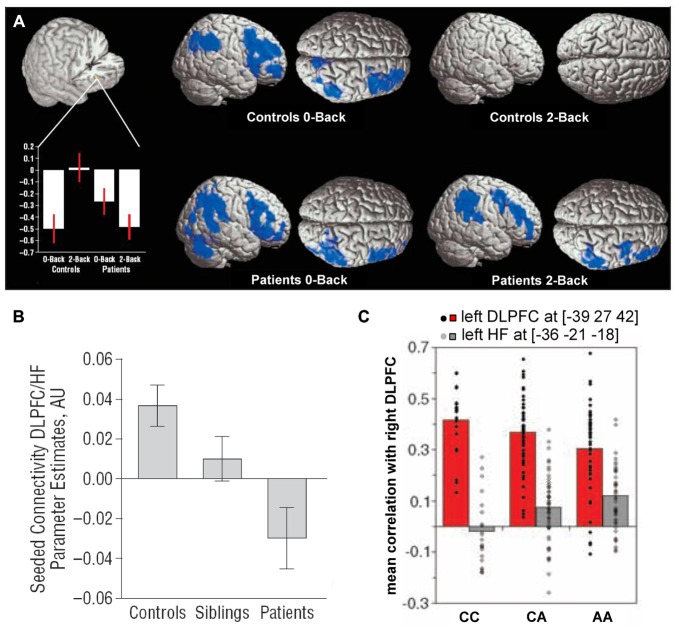
**Hippocampal-prefrontal interactions in schizophrenia patients and at-risk individuals. (A)** Hippocampal-prefrontal functional connectivity during working memory in schizophrenia. Bottom left: connectivity of the dorsolateral PFC with the hippocampus (cutout above) during performance of an n-back task. During a control condition (0-back), connectivity was negative in controls and patients. However, during the working memory condition (2-back) connectivity was absent in controls but persisted in patients. Voxels highlighted in blue indicate regions showing negative functional connectivity with the hippocampus. Adapted from Meyer-Lindenberg et al. (2005). **(B)** Connectivity between the hippocampus and PFC in schizophrenia patients, their unaffected siblings and control subjects. Note that the connectivity in siblings is intermediate between that of patients and controls. Adapted from Rasetti et al. (2011). **(C)** Hippocampal-prefrontal connectivity in carriers of a schizophrenia risk allele in the gene *ZNF804A*. Connectivity between the right dorsolateral PFC and left hippocampus (gray bars) increases with the number of risk allelels (A). Also shown is connectivity with the contralateral PFC (red bars). Adapted from Esslinger et al. (2009).

Early studies comparing the hippocampus and dorsolateral PFC in schizophrenia patients suggested that connectivity between them might be disrupted (Weinberger et al., 1992; Fletcher, 1998; Heckers et al., 1998). Several subsequent studies directly examined functional connectivity between the two regions in patients performing working memory tasks. Meyer-Lindenberg et al. (2005) found that the hippocampus and dorsolateral PFC were uncoupled during an n-back working memory task in healthy subjects but showed negative functional connectivity (anticorrelation) in patients (Figure 3A). Similar results were obtained by Rasetti et al. (2011), although here hippocampal-prefrontal connectivity was positive in healthy subjects but negative in patients. Henseler et al. (2010) also observed reduced connectivity and found that patients with weaker connectivity displayed worse performance on a verbal working memory task as well as more severe disease symptoms. A study measuring effective connectivity furthermore suggested a decrease in the influence of the hippocampus over the PFC in schizophrenia patients (Benetti et al., 2009). Reduced resting state functional connectivity was also observed between the hippocampus and the mPFC (Zhou et al., 2008). Notably, this study also observed structural abnormalities in the fornix, the fiber bundle that connects the hippocampus to neocortical areas, including the PFC.

Hippocampal-prefrontal interactions have also been examined in healthy individuals that are at increased risk for developing schizophrenia based either on psychological assessment or from having a sibling with the disease. These studies have found hippocampal-prefrontal interactions in at-risk individuals to be either similar to patients (Benetti et al., 2009) or intermediate between patients and controls (Rasetti et al., 2011; Figure 3B). The fact that siblings of schizophrenia patients show abnormal hippocampal-prefrontal interactions suggests a genetic contribution. Recently, genome-wide association studies have identified several common single-nucleotide polymorphisms (SNPs) that are associated with increased risk for developing schizophrenia (Harrison, 2015) and the impact of these SNPs on brain function has now been examined in several studies (Gurung and Prata, 2015). One well-studied SNP occurs in the *ZNF804A* gene that encodes for a zinc finger protein which acts as a transcription factor, although its exact function is not well understood (O’Donovan et al., 2008). Several studies have found that hippocampal-prefrontal functional connectivity during working memory increases with the number of *ZNF804A* risk alleles carried by an individual (Figure 3C; Esslinger et al., 2009; Rasetti et al., 2011; Paulus et al., 2013; Cousijn et al., 2015). This effect, however, appears to be state-dependent since it is not seen during other tasks (Esslinger et al., 2011). Interestingly, theta oscillations, which are important for hippocampal-prefrontal synchrony (Colgin, 2011), are also decreased in carriers of the risk allele (Cousijn et al., 2015). A schizophrenia-associated SNP in the gene coding for the alpha 1C subunit of the L-type voltage-gated calcium channel (*CACNA1C*) is also associated with increased hippocampal-prefrontal connectivity (Paulus et al., 2014). Finally, an SNP in the gene coding for microRNA 137 (*MIR137*) was associated with weaker negative functional connectivity between the hippocampus and PFC at rest (Liu et al., 2014).

## Hippocampal-Prefrontal Interactions in Animal Models of Schizophrenia

In addition to patient studies, animal models of schizophrenia are likely to play an important complementary role in elucidating the underlying pathophysiology of the illness. Given the complexity and diversity of schizophrenia symptoms, it is unrealistic to expect such models to capture the disease in its entirety and any model will inevitably be a partial one. Animal models of schizophrenia differ in their “construct validity”, that is how well they model the known causes and risk factors of the illness (Nestler and Hyman, 2010). Mouse models carrying genetic risk factors, especially ones associated with a large increase in disease risk, can be considered to have good construct validity. Animals can also be exposed to some of the environmental risk factors for the disease. Putative pathophysiological mechanisms based on known abnormalities in patients (f.ex. an increase or a decrease in the function of certain neurotransmitter receptors) can be recreated in animal models and they can also be used to study the consequences of neurodevelopmental perturbations. In all animal models the effects of these manipulations on brain structure, brain function and behavior can be experimentally assessed; importantly, they allow the use of invasive methods that can examine neural circuit function in much greater detail than is possible in patient studies (Sigurdsson, 2015). Typically, animal models of schizophrenia display abnormalities that resemble some of the manifestations of the illness in humans, what is often referred to as “face validity” (Nestler and Hyman, 2010). Although animal models cannot display symptoms like hallucinations and delusions, they can exhibit some of the so-called “endophenotypes” of the disease: abnormalities that are reliably found in schizophrenia patients although they are not part of the diagnostic criteria of the illness (Amann et al., 2010). For example, schizophrenia patients show impaired “prepulse inhibition” (PPI), a measure of sensorimotor gating, that is also seen in many animal models of the illness (Powell et al., 2012). Deficits in cognitive functions such as working memory can also be revealed in animal models (Arguello and Gogos, 2010). Endophenotypes can also encompass abnormalities in brain structure (e.g., enlarged ventricles) or function, including disruptions in inter-areal interactions (Tost et al., 2012; Rosen et al., 2015).

**Figure 4:**
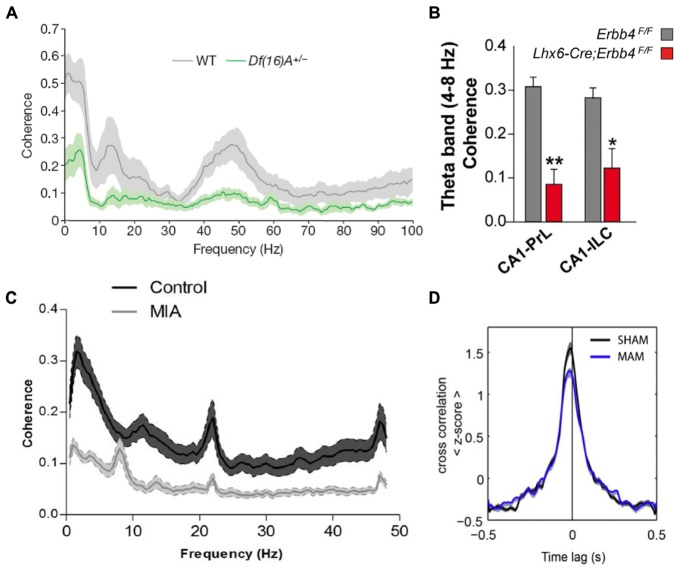
**Hippocampal-prefrontal interactions in animal models of schizophrenia.** A reduction in hippocampal-prefrontal synchrony is seen across different animal models of schizophrenia representing diverse risk factors and pathophysiological mechanisms.** (A)** Decreased hippocampal-prefrontal local field potential (LFP) coherence in *Df(16)A*^+/−^ mice, which model the human 22q11.2 microdeletion, during a SWM task (Sigurdsson et al., 2010). **(B)** Decreased hippocampal-prefrontal theta coherence in mice lacking the *ErbB4* receptor on parvalbumin interneurons (Lhx6-Cre; *Erbb4*F/F; Del Pino et al., 2013). **(C)** Decreased hippocampal-prefrontal LFP coherence in offspring of maternal immune activation (MIA)-treated rats (Dickerson et al., 2010). **(D)** Reduced cross-correlation between hippocampal-prefrontal cell pairs in offspring of methylazoxymethanol acetate (MAM)-treated rats, a neurodevelopmental model of schizophrenia, during slow-wave sleep (Phillips et al., 2012).

Because of their central role in schizophrenia pathophysiology, many of the studies that have examined brain function in animal models have focused on the hippocampus and the PFC and revealed abnormalities in synaptic plasticity, inhibitory transmission and neural synchrony in the two regions (Crabtree and Gogos, 2014; Rosen et al., 2015; Sigurdsson, 2015). Impaired hippocampal-prefrontal synchrony has also been observed in many different models. This was first demonstrated in a genetic mouse model of the 22q11.2 microdeletion, which is associated with a 30-fold increase in the risk for developing schizophrenia (Karayiorgou et al., 2010). *Df(16)A*^+/−^ mice, which lack the critical genes affected by the 22q11.2 microdeletion, display several behavioral deficits, including impairments in working memory (Stark et al., 2008; Drew et al., 2011). To examine what neural circuit abnormalities could contribute to these working memory deficits, Sigurdsson et al. (2010) recorded from the hippocampus and PFC of *Df(16)A*^+/−^ mice while they performed a SWM task. Reduced phase locking of prefrontal neurons to hippocampal theta oscillations was observed in these mice, as well as reduced LFP coherence between the two structures (Figure 4A). Importantly, theta synchrony within either the hippocampus or the PFC was not affected, suggesting a selective disruption of long-range synchrony. The synchrony deficits were also correlated with the working memory impairments: theta synchrony prior to training predicted the number of trials needed to learn the working memory task and also developed more slowly in the *Df(16)A*^+/−^ mice during learning of the task.

Subsequent studies have replicated and extended these findings in different genetic models. One of the outstanding questions of the Sigurdsson et al. (2010) study is which of the roughly 30 genes affected by the 22q11.2 microdeletion is responsible for the hippocampal-prefrontal synchrony impairment. Recently, Mukai et al. (2015) have addressed this by examining mice with a deletion in a single 22q11.2 gene, *Zdhhc8*. These mice also showed deficits in hippocampal-prefrontal synchrony, which were correlated with their working memory impairments. Another important finding in this study was that in *Zdhhc8* mice the projections of ventral hippocampal neurons formed fewer branches within the PFC, suggesting a possible anatomical basis for the synchrony deficits. Variants in the gene coding for Neuregulin 1 (*NRG1*) have also been strongly associated with increased risk for schizophrenia (Harrison and Law, 2006). *NRG1* is a signaling molecule that binds to the *ErbB4* receptor and is important for neural development (Mei and Nave, 2014). Interestingly, the *ErbB4* receptor is found almost exclusively on parvalbumin-positive (PV) interneurons (Fazzari et al., 2010), which have consistently been implicated in the pathophysiology of schizophrenia (Volk and Lewis, 2010). In mice lacking the *ErbB4* receptor on PV interneurons hippocampal-prefrontal synchrony is reduced, and local gamma synchrony within the two structures is increased (Figure 4B; Del Pino et al., 2013). Another genetic mutation that has received a great deal of attention occurs in the gene Disrupted-in-Schizophrenia 1 (*DISC1*) and was found to co-segregate with psychiatric illness including schizophrenia, bipolar disorder and depression in a large Scottish family (St Clair et al., 1990; Millar et al., 2000). Although its function is not well understood, the *DISC1* protein appears to play a role in neural development and several animal models have been developed to understand the functional consequences of mutations within the *DISC1* gene (Brandon and Sawa, 2011). In one of these models, in which mice overexpress the mutated *DISC1* gene, hippocampal-prefrontal synchrony was found to be normal (Sauer et al., 2015). Interestingly, these mice did not show working memory deficits but instead displayed a behavioral phenotype more consistent with depression. However, another study using the same model found deficits in synaptic plasticity at ventral hippocampal inputs to the PFC *in vitro* (Dawson et al., 2015).

Environmental events can also significantly increase risk for schizophrenia (McDonald and Murray, 2000). One well-established example is viral infection during pregnancy, which increases risk for schizophrenia in the offspring (Canetta and Brown, 2012). Activation of the mother’s immune system appears to be the responsible causal factor which has led to the use of maternal immune activation (MIA) as a model of environmental risk for schizophrenia (Shi et al., 2003; Dickerson and Bilkey, 2013). In the offspring of MIA-treated rats, hippocampal-prefrontal synchrony was found to be impaired and the synchrony deficits correlated with impairments in PPI (Figure 4C; Dickerson et al., 2010). In a follow-up study, the synchrony impairment was found to occur selectively between the PFC and the dorsal, but not the ventral, hippocampus (Dickerson et al., 2014). Theta synchrony deficits in MIA animals could also be ameliorated by the administration of the antipsychotic clozapine (Dickerson et al., 2012). To examine how this environmental risk factor interacts with genetic risk factors, Hartung et al. (2014) exposed mice carrying a mutation in the *DISC1* gene to MIA. Interestingly, this caused a disruption in hippocampal-prefrontal synchrony during early development (postnatal day 8–10) whereas exposure to only one of the two risk factors did not have an effect, consistent with a role for gene-environment interactions in the pathophysiology of schizophrenia.

Multiple lines of evidence indicate that schizophrenia is a neurodevelopmental disorder (Lewis and Levitt, 2002). Several animal models of schizophrenia have therefore been developed to examine the consequences of neurodevelopmental perturbations on brain function and behavior in the adult. Two widely used models involve administering the mitotoxin methylazoxymethanol acetate (MAM) during gestation (Moore et al., 2006) or lesioning the vHPC in neonatal animals (NVHL; Lipska et al., 1993). Neither the MAM nor the NVHL model reflect known developmental perturbations that have been associated with schizophrenia. However, in both models behavioral and neural abnormalities, including deficits in PPI and working memory, emerge in adulthood, mirroring the onset of symptoms in humans (Lodge and Grace, 2009; O’Donnell, 2012). These models can therefore help to illuminate how neurodevelopmental perturbations leading to schizophrenia-like symptoms in adulthood affect the functioning of neural circuits. Impaired hippocampal-prefrontal interactions are seen in both these models. Phillips et al. (2012) recorded from the hippocampus and PFC in MAM animals during sleep and found reduced hippocampal-prefrontal synchrony both in measures of LFP coherence as well as in the cross-correlations of neurons recorded in both structures (Figure 4D). These results are interesting in light of the fact that sleep abnormalities are observed in schizophrenia patients (Gardner et al., 2014) and that oscillations during sleep are important for memory consolidation. Lee et al. (2012) recorded from the hippocampus and PFC of adult NVHL rats during a spatial task that required animals to pay attention to distal cues while ignoring local cues in their environment. Intra-hippocampal synchrony was greater during performance on this task and was reduced in NVHL animals together with impaired behavioral performance. Hippocampal-prefrontal synchrony, although not modulated by the task, was also reduced. Notably, the effect of NVHL on synchrony and behavior could be prevented if animals received cognitive training during adolescence, an intriguing result with potential therapeutic implications.

Antagonists of the N-methyl-D-aspartate receptor (NMDAR) such as ketamine can induce symptoms reminiscent of schizophrenia in healthy individuals and are frequently used as a pharmacological model of the disease in both humans and animals (Javitt and Zukin, 1991; Frohlich and van Horn, 2014). A recent study found that ketamine increased hippocampal-prefrontal functional connectivity measured using fMRI in anesthetized rats (Gass et al., 2014). A follow-up study by the same group replicated this result and furthermore showed that ketamine had the same effect in healthy human subjects (Grimm et al., 2015). Although there were methodological differences between the rat and human experiments (rats were anesthetized whereas human subjects were awake and also received a lower dose of ketamine) the results are notable for the fact that measures of hippocampal-prefrontal interactions could be directly compared between species, which is usually not possible (an issue discussed further below).

## Emerging Themes and Outstanding Questions for Future Research

### How Do Hippocampal-Prefrontal Interactions Support Cognition and Behavior?

The studies reviewed above demonstrate that hippocampal-prefrontal interactions are observed during a variety of behavioral and cognitive tasks and are dynamically modulated by task demands. Lesion studies and optogenetic manipulations furthermore suggest that these interactions are necessary for behavioral performance in some tasks. Yet exactly how hippocampal-prefrontal interactions support cognition and behavior is not fully understood and an important goal of future studies will be to deepen our understanding in this respect. This is not only an important goal in its own right but may also aid in understanding the consequences of dysfunctional interactions in psychiatric illness and animal disease models. One possibility is that hippocampal-prefrontal interactions, and perhaps inter-areal interactions in general, support behavior by allowing information to be relayed between brain regions. Consistent with this, several studies have found that the degree to which prefrontal neurons are phase-locked to hippocampal oscillations is predictive of their response properties. For example, prefrontal neurons that were phase-locked to theta oscillations in the dHPC were more likely to predict animals’ upcoming choice in a T-maze working memory task than cells that were not phase-locked (Fujisawa and Buzsáki, 2011; see also Hyman et al., 2011; Remondes and Wilson, 2013). Another study found that phase-locking to ventral, but not dorsal, hippocampal theta oscillations was associated with stronger anxiety-related firing patterns (Adhikari et al., 2011). One interpretation of these findings is that neurons’ firing properties are influenced by the inputs (direct or indirect) that they receive from other brain regions, which in turn is reflected in their synchronization to neural activity in those regions. Consistent with this idea, silencing ventral hippocampal inputs to PFC abolishes the goal-specific firing of PFC neurons in the sample phase of a SWM task, as well as their phase locking to ventral hippocampal gamma (but not theta) oscillations (Spellman et al., 2015). This suggests that ventral hippocampal inputs transmit—perhaps via hippocampal-prefrontal gamma synchrony—spatial information to the PFC that might help it to encode previously visited locations. In future studies, similar approaches could help reveal what kinds of information are conveyed by hippocampal inputs during other behaviors, for example anxiety. Also important will be to better understand how the PFC influences activity in the hippocampus, which has been relatively less well studied. Inactivation studies suggest that the PFC modulates the activity of hippocampal place cells during memory-guided tasks (Navawongse and Eichenbaum, 2013) which could be mediated by direct projections from PFC to hippocampus (Rajasethupathy et al., 2015). Interestingly, these projections preferentially target highly connected neurons (or “hubs”) within the hippocampal network that emerge following learning and may be critical for memory retrieval (Rajasethupathy et al., 2015).

Ultimately, hippocampal-prefrontal interactions need to be understood in terms of their contribution to behavior. As the study of Spellman et al. (2015) elegantly demonstrates, optogenetic methods are now poised to address this question in a temporally and pathway-specific (and potentially cell-specific) manner. Although this study shows that direct inputs from the hippocampus to the PFC are required during the “sample” phase of a SWM task, it remains unclear which inputs are necessary during the “choice” phase of these tasks. Given that the choice phase is associated with enhanced theta synchrony, which is not affected by silencing of direct hippocampal inputs, it could rely more on indirect inputs (see below). Also important will be to examine the causal contribution of hippocampal-prefrontal interactions to other cognitive and behavioral functions such as anxiety (Adhikari et al., 2010) and memory consolidation (Wierzynski et al., 2009). The different roles of the dorsal and ventral poles of the hippocampus will need to be explored as well as whether different cell populations in the two structures support synchrony during different behavioral tasks. Hippocampal-prefrontal interactions should also be examined in the broader context of other brain networks. For example, slow oscillations in the ventral tegmental area coordinate the activity of both hippocampus and PFC during working memory (Fujisawa and Buzsáki, 2011). Entorhinal inputs to the hippocampus are also necessary for SWM (Yamamoto et al., 2014) and both the hippocampus and the PFC cooperate with the amygdala to control fear and anxiety (Popa et al., 2010; Lesting et al., 2013; Likhtik et al., 2014). An integrative view of how the hippocampal-prefrontal circuit operates within these larger networks will therefore be required.

Finally, it will be important to better understand the mechanisms underlying hippocampal-prefrontal interactions. As already discussed, inter-areal synchrony is often observed in the form of coordinated oscillations in different brain regions. However, although the mechanisms underlying these oscillations have in some cases been elucidated (Buzsáki, 2002; Buzsáki and Wang, 2012), their inter-areal coordination is less well understood. Do they reflect the monosynaptic influence of one brain area on another, or indirect polysynaptic pathways or perhaps the mutual influence of a third region on the other two? In agreement with the first possibility, optogenetically silencing the projections from the vHPC to the PFC reduces gamma synchrony between the two regions (Spellman et al., 2015). Similarly, genetic silencing of monosynaptic inputs from the entorhinal cortex to the CA1 region of the hippocampus reduces gamma synchrony between them (Yamamoto et al., 2014). These results suggest that gamma synchrony reflects the strength of synaptic drive from direct afferent inputs. However, it is also noteworthy that in both of the aforementioned studies, synchrony in the theta frequency range was unaffected. This could mean that inter-areal theta synchrony requires additional brain regions that act as relay stations or provide common input. For example, the medial septum provides rhythmic theta-frequency input to both the entorhinal cortex and the hippocampus (Buzsáki, 2002) and thus likely contributes to theta synchrony between them. Pharmacological inactivation of the vHPC suggests that it is important for synchronizing theta oscillations in the PFC and the dHPC (O’Neill et al., 2013). Other structures that are connected with both the hippocampus and the PFC and could mediate synchrony between them, as already mentioned, include the NR of the thalamus (Vertes, 2006; Cassel et al., 2013) and the lateral entorhinal cortex (Moser et al., 2010). Notably, inactivating the NR causes impairments in SWM (Layfield et al., 2015). Whether inactivating these structures disrupts hippocampal-prefrontal synchrony will be an important question for future studies. Understanding the mechanisms underlying hippocampal-prefrontal synchrony will be especially important for interpreting synchrony deficits in animal disease models.

### How Do Disrupted Hippocampal-Prefrontal Interactions Contribute to Psychiatric Disease?

Altered hippocampal-prefrontal connectivity has been consistently observed in studies of both schizophrenia patients and individuals at risk for the disease, making it a strong candidate as an “intermediate phenotype” between the causes of the disease and its symptoms (Meyer-Lindenberg and Weinberger, 2006; Tost et al., 2012). Yet exactly how this abnormality manifests itself is not always consistent across studies. Most studies in patients have found a reduction in positive functional connectivity (e.g., Zhou et al., 2008), but some studies have found increased negative functional connectivity (i.e., anti-correlated activity) between hippocampus and PFC (e.g., Meyer-Lindenberg et al., 2005). Studies in at-risk individuals, in particular those carrying genetic risk variants, have also found that hippocampal-prefrontal connectivity increases with the number of risk alleles (e.g., Esslinger et al., 2009). Differences in experimental conditions across studies could contribute to these discrepancies. Notably, Esslinger et al. (2011) found increased negative connectivity in at-risk individuals during performance of the n-back working memory task but not during an emotion recognition task, suggesting that functional connectivity deficits can be task-specific. An important complementary approach should therefore be to measure functional connectivity in subjects at rest (e.g., Zhou et al., 2008), which could facilitate comparisons across different studies. Deficits in resting state functional connectivity, which is more reflective of the brain’s intrinsic anatomical connectivity (Buckner et al., 2013), could also be more directly related to structural connectivity deficits seen in schizophrenia patients (Pettersson-Yeo et al., 2011).

Another important outstanding question is how alterations in hippocampal-prefrontal connectivity contribute to the symptoms of schizophrenia. Some of the studies reviewed here have reported a correlation between hippocampal-prefrontal connectivity and disease symptoms as well as cognitive performance (Henseler et al., 2010). Abnormal connectivity between frontal and temporal lobes has also been found to correlate with hallucinations in schizophrenia patients (Lawrie et al., 2002). Nevertheless, the relationship between connectivity impairments and disease symptoms or cognitive deficits remains underexplored and will require more attention in future studies. A better understanding of the role of hippocampal-prefrontal interactions in the healthy brain, as discussed above, will likely be essential in formulating specific disease-relevant hypotheses. It will also be important to consider that abnormal hippocampal-prefrontal interactions likely contribute to psychiatric illnesses other than schizophrenia. Given the role of the hippocampal-prefrontal circuit in fear and anxiety, as discussed above, it is not surprising that abnormalities in this circuit are beginning to be revealed in anxiety and mood disorders (Godsil et al., 2013; Genzel et al., 2015; Li et al., 2015). It is also interesting to note that many of the SNPs that disrupt hippocampal-prefrontal connectivity increase the risk not only for schizophrenia but also other psychiatric diseases such as bipolar disorder (Gurung and Prata, 2015; Harrison, 2015). The hippocampal-prefrontal circuit is also vulnerable to stress, which is a common risk factor for many psychiatric disorders (Godsil et al., 2013). Disrupted hippocampal-prefrontal interactions may therefore be a fundamental deficit underlying multiple psychiatric diseases, although exactly how they contribute to their psychopathology remains to be determined.

The hippocampal-prefrontal circuit has begun to be investigated in animal models of schizophrenia and the results have been in general agreement with those of patient studies in revealing disrupted interactions between the two structures. Nevertheless, there are discrepancies between these two lines of research that make direct comparisons difficult and which future studies should attempt to address. One notable difference is that studies in animal models have examined connectivity with the mPFC whereas patient studies have focused almost exclusively on the dorsolateral PFC, which plays a key role in the cognitive functions that are disrupted in the disease. Although the rodent mPFC subserves some of the same cognitive functions as the primate dorsolateral PFC some of its subregions also bear a strong resemblance to the mPFC of primates both in terms of behavior and anatomy (Condé et al., 1995; Öngür and Price, 2000). Notably, both the rodent and primate mPFC receives direct synaptic input from the ventral (or anterior) hippocampus (Rosene and Van Hoesen, 1977; Barbas and Blatt, 1995; Hoover and Vertes, 2007). In human subjects at rest, the hippocampus is positively correlated with the mPFC, consistent with the anatomy (Vincent et al., 2006). Examining hippocampal-mPFC coupling in more detail in patients (e.g., Zhou et al., 2008) will therefore be important in future studies. Also important will be to examine connectivity under similar behavioral conditions in patients and animal models. Many of the cognitive tasks used in patient studies, such as the n-back task, are difficult to replicate in animals although virtual reality versions of spatial tasks used in rodents can be used to study hippocampal-prefrontal interactions in humans (Bähner et al., 2015). Resting state measurements in patients could also be more directly compared with similar measurements made in animal models. Further strategies to reconcile patient and animal model studies will be discussed in the following section.

### What can We Learn from Animal Models about Disruptions in Hippocampal-Prefrontal Interactions?

The studies reviewed here show that disrupted hippocampal-prefrontal interactions are commonly observed across different animal models of schizophrenia. In contrast to the somewhat variable nature of impairments in schizophrenia patients, reduced synchrony between the two structures has consistently been observed in animal models. This impairment has been observed under a variety of behavioral conditions (anesthesia, sleep, wakefulness and during cognitive tasks) and using a number of different analytical methods (cross-correlations, spike-LFP phase-locking and LFP coherence). The results of these studies, together with the evidence from studies in patients and at-risk individuals reviewed here, lend further support to the notion that disrupted hippocampal-prefrontal interactions may be a fundamental pathophysiological mechanism underlying schizophrenia. Going beyond the results of patient studies, studies in animal models have related disrupted hippocampal-prefrontal interactions to a number of risk factors (both genetic and environmental) and pathophysiological mechanisms of the illness and demonstrated synchrony impairments at the cellular level. However, it is also important to point out that although most studies have focused on the hippocampus and PFC, disrupted interactions are also observed between other structures in animal models (reviewed in Sigurdsson, 2015), as well as in schizophrenia patients (Pettersson-Yeo et al., 2011).

An important next step will be to use animal models to understand the exact mechanisms that lead to disruptions in hippocampal-prefrontal interactions. The diverse array of manipulations used in the different animal models reviewed here (i.e., genetic, environmental, neurodevelopmental) raises the question whether they all ultimately converge on a common causal mechanism to disrupt hippocampal-prefrontal synchrony. In this respect, it is interesting to note that many of the genetic risk factors for schizophrenia affect genes that play important roles in neural development, including the development and plasticity of synaptic connections. It is therefore tempting to speculate that disruptions in long-range synaptic connections between the hippocampus and PFC might be responsible for impairments in hippocampal-prefrontal synchrony. Supporting this possibility is the recent study by Mukai et al. (2015) demonstrating abnormalities in the projections of hippocampal neurons to the PFC in *Zdhhc8*-deficient mice. Whether similar anatomical impairments are found in other models displaying reduced hippocampal-prefrontal synchrony remains to be determined. Relating synchrony deficits to anatomical impairments is an important step towards understanding their possible molecular mechanisms, which can lead to potential targets for novel treatments. Notably, Mukai et al. (2015) were able to show that the structural deficits in hippocampal-prefrontal projections in *Zdhhc8*-deficient mice were due to abnormalities in protein regulation that, when reversed, could rescue the structural deficits. Whether similar manipulations can also rescue the deficits in synchrony is an interesting question for future research.

How disrupted hippocampal-prefrontal synchrony affects cognition and behavior in animal models of schizophrenia needs to be better understood. Consistent with its role in working memory, as reviewed above, deficits in hippocampal-prefrontal synchrony have been found to correlate with the degree of working memory impairments in some animal models (Sigurdsson et al., 2010; Mukai et al., 2015). Correlations with impairments in PPI have also been observed (Dickerson et al., 2010). Whether hippocampal-prefrontal synchrony deficits are associated with other impairments seen in animal models, for example in attentional set-shifting or long-term memory, will need to be investigated. Interestingly, some studies have reported selective impairments in synchrony between the PFC and either the dorsal (Dickerson et al., 2014) or the ventral (Mukai et al., 2015) hippocampus. Given that the dorsal and ventral poles have different behavioral functions (Bannerman et al., 2004) it is worth asking whether deficits in their synchronization with the PFC have differential effects on behavior. It will also be important to go beyond correlative results and establish causal relationships between synchrony deficits and behavioral impairments. Rescuing behavioral impairments by reversing hippocampal-prefrontal synchrony deficits, perhaps by targeting relevant molecular mechanisms (e.g., Mukai et al., 2015), would constitute powerful evidence in this respect. Reproducing synchrony deficits in healthy animals, for example using optogenetic manipulations (e.g., Spellman et al., 2015), will also be an important complementary strategy.

Another important goal of future research should be to better integrate findings from animal models and patient studies. Although both kinds of studies have revealed disruptions in hippocampal-prefrontal interactions, their results are not always directly comparable, as already discussed. In addition to the reasons already mentioned is the fact that the methods used to measure interactions in animal models and patients differ considerably. In both situations, interactions are quantified by correlating fluctuations in neural activity in the hippocampus and PFC; however, these fluctuations occur on a timescale of milliseconds when measured electrophysiologically in animal models whereas in fMRI measurements in patients they occur at a timescale of tens of seconds (Fox and Raichle, 2007). Because of this difference in timescales, it is far from clear how measures of functional connectivity in patients relate to measures of neural synchrony in animal models. One way to address this could be to use measures from electrophysiological data that better correspond with the fMRI BOLD signal. This signal is known to closely correlate with the power of local gamma oscillations (Shmuel and Leopold, 2008) and the amplitude of these oscillations fluctuates with a slow time course similar to that of the BOLD signal. Correlations in gamma power fluctuations across brain regions can therefore provide a measure of functional connectivity similar to that obtained using fMRI (Schölvinck et al., 2010) and could be applied to animal models in the future. Another approach could be to measure functional connectivity in animal models using fMRI. This approach has already begun to be used (e.g., Grimm et al., 2015) although to date it has been restricted to anesthetized animals. Combining fMRI with simultaneous electrophysiological measurements (Logothetis et al., 2001) could prove especially fruitful for relating neural synchrony to functional connectivity. Finally, the discovery of SNPs reliably associated with schizophrenia (Harrison, 2015) should make it easier to examine the impact of genetic risk variants in both humans and animal models.

## Concluding Summary

Considerable progress has been made in delineating the functions of different brain regions yet how their activity is coordinated and integrated to produce adaptive behavior is less well understood. The studies reviewed here have provided insights into how interactions between two specific brain regions, the hippocampus and PFC, contribute to various aspects of cognition and behavior. Electrophysiological studies have revealed how hippocampal-prefrontal interactions are mediated by synchronized neural activity in the two regions, and how these interactions are dynamically modulated by behavioral demands. Lesion studies and optogenetic manipulations have furthermore demonstrated the causal relevance of hippocampal-prefrontal interactions to behavior. Collectively, these findings have implicated hippocampal-prefrontal interactions in cognitive processes such as SWM as well as emotional behaviors such as fear and anxiety. Studies have also begun to reveal how these interactions influence neural representations and activity patterns in the two regions, which should be an important focus of future research. In addition to being involved in normal brain function, there is also evidence that hippocampal-prefrontal interactions are disrupted in psychiatric illness. This has been most consistently demonstrated for schizophrenia, which we have focused on in this review, although similar deficits may be found in other psychiatric diseases as well. Studies in animal models of schizophrenia have demonstrated that hippocampal-prefrontal interactions can be disrupted by specific risk factors and pathophysiological mechanisms of the disease and have also begun to uncover the cellular and circuit mechanisms underlying these impairments. However, although disruptions in hippocampal-prefrontal interactions likely contribute to deficits in cognition, it is not yet clear how they might lead to disease symptoms. A deeper understanding of the pathophysiological significance of these disturbances will likely require further knowledge of how hippocampal-prefrontal interactions operate in the healthy brain.

## Author Contributions

TS and SD wrote the manuscript.

## Conflict of Interest Statement

The authors declare that the research was conducted in the absence of any commercial or financial relationships that could be construed as a potential conflict of interest.
